# Risk factors associated with lymph node metastasis in papillary thyroid cancer: a retrospective analysis based on 2,428 cases

**DOI:** 10.3389/fonc.2024.1473858

**Published:** 2024-11-06

**Authors:** Kejie Yu, Xianjiang Wu, Lei Dai, Qi Le, Yue Xie, Yingchun Wang, Weidong Zhang

**Affiliations:** Department of Thyroid Surgery, Ningbo No.2 Hospital, Ningbo, Zhejiang, China

**Keywords:** papillary thyroid cancer, central lymph node metastasis, lateral lymph node metastasis, skip metastasis, risk factor

## Abstract

**Background:**

Papillary thyroid cancer (PTC) with lymph node metastasis (LNM) is associated with an increased risk of postoperative recurrence. Understanding the clinical characteristics of PTC patients can help surgeons assess the likelihood of LNM. This study aims to identify risk factors for LNM in PTC patients.

**Methods:**

We retrospectively analyzed clinical data from 2,428 patients diagnosed with PTC who underwent initial thyroid surgery in our single center. Risk factors related to central lymph node metastasis (CLNM), lateral lymph node metastasis (LLNM), and skip metastasis were investigated by univariate and multivariate binary logistic regression analyses.

**Results:**

Univariate analysis revealed that the following factors were associated with an increased likelihood of CLNM (P<0.05): male gender, age < 45 years, maximal axial diameter (MAD) > 1 cm, body mass index (BMI) ≥ 28 kg/m² and multifocality. Univariate analysis also revealed that patients with age < 45 years, MAD > 1 cm, BMI ≥ 28 kg/m², multifocality, and CLNM demonstrated a higher propensity for LLNM (P<0.05). Lower pole tumors were more likely to have CLNM than upper pole tumors, while upper pole tumors were more prone to present LLNM (P<0.05) and skip metastasis (P<0.05). Multivariable binary logistic regression analysis identified that gender (odds ratio [OR], 1.732; 95% CI, 2.113–2.577; P < 0.001), age (OR, 1.905; 95% CI, 1.596–2.273; P < 0.001), MAD (OR, 4.639; 95% CI, 3.639–5.913; P < 0.001), and multifocality (OR, 1.860; 95% CI, 1.453–2.381; P < 0.001) were independent risk factors for CLNM and MAD (OR, 5.289; 95% CI, 3.777–7.404; P<0.001), multifocality (OR, 1.858; 95% CI, 1.248–2.766; P=0.002), and CLNM (OR, 5.030; 95% CI, 3.347–7.561; P<0.001) for LLNM.

**Conclusion:**

Despite the overall postoperative recurrence rate in PTC patients is low, identifying risk factors such as male gender, age < 45 years, MAD > 1 cm, multifocality, and CLNM can help predict LNM. In specific cases, selective lymphadenectomy in the central or lateral neck area may be warranted.

## 1 Introduction

In recent years, the global incidence of thyroid cancer has seen a steady increase, making it the most prevalent malignant tumor of the endocrine system (1). Notably, the number of new thyroid cancer cases worldwide rose from approximately 122,800 in 2000 to 586,202 by 2020 (2, 3). Among malignant thyroid tumors, over 90% are classified as papillary thyroid cancer (PTC), which primarily spreads through lymph node metastasis (LNM) (4, 5). LNM typically begins in the central region before progressing to the lateral neck area (6), although isolated cases of skip metastasis (lateral neck metastasis without central involvement) also occur (7, 8).Research has highlighted the correlation between LNM in the neck and an elevated risk of post-surgery recurrence (9). Additionally, LNM in the lateral neck region often indicates a poor prognosis and an increased likelihood of requiring additional surgical intervention (10, 11).

However, controversy surrounds the routine use of central lymph node dissection (CLND) during PTC surgery due to the associated risks, including laryngeal nerve injury and hypoparathyroidism (12, 13). The American Thyroid Association (ATA) recommends therapeutic CLND for patients with clinical evidence of lymph node involvement (14). Identifying risk factors associated with LNM is crucial for surgeons to accurately assess the likelihood of lymph node involvement in patients. This knowledge enables more precise surgical planning and informed decision-making regarding neck lymph node dissection (LND) (15, 16). Despite ongoing debate in clinical practice, this retrospective study specifically examines the correlations between various clinical characteristics and central lymph node metastasis (CLNM), lateral lymph node metastasis (LLNM), and skip metastasis. The findings aim to facilitate the identification of risk factors for LNM and provide guidance for clinical decision-making.

## 2 Materials and methods

### 2.1 Patients and clinical data collection

We conducted a retrospective analysis of 2,428 patients diagnosed with PTC who underwent their initial thyroid surgery at Ningbo NO. 2 Hospital between January 2023 and December 2023. The study included patients who met the following criteria:

- Underwent their first thyroid surgery
- Possessed comprehensive clinical data
- Received postoperative pathology confirmation of PTC without concurrent other types of thyroid cancer

Clinical and pathological characteristics, including gender, age, maximal axial diameter (MAD), body mass index (BMI), multifocality, Hashimoto’s thyroiditis (HT), CLNM, LLNM, and tumor location, were extracted from electronic medical records and the Ningbo Resident Health Big Data Archive. Continuous variables (age, MAD, BMI) were categorized based on predefined cut-off points:

- Age: ≥45 years and <45 years
- MAD: >1 cm and ≤1 cm
- BMI: ≥28 kg/m² (obese) and <28 kg/m² (non-obese)

We categorized tumor location as upper pole, middle pole, and lower pole (excluding scattered and isthmic tumors). The diagnosis of HT and LNM relied on postoperative histopathological examination of tissue samples.

### 2.2 Statistical analysis

Data management and statistical analyses were performed using IBM SPSS Statistics, Version 27.0 (SPSS Inc., Chicago, IL, USA). Categorical variables were presented as frequencies and percentages. Univariate analysis utilized the chi-square test, with P < 0.05 considered statistically significant. Variables identified through univariate analysis underwent multivariate binary logistic regression to assess the impact of potential risk factors. Results were reported as odds ratios (ORs) with 95% confidence intervals (CIs). A significance level of P<0.05 was considered statistically significant.

## 3 Results

This retrospective analysis included 2,428 patients diagnosed with PTC, of whom 944 (38.88%) exhibited CLNM and 177 (7.23%) presented with LLNM.

### 3.1 CLNM

**Figure 1:**
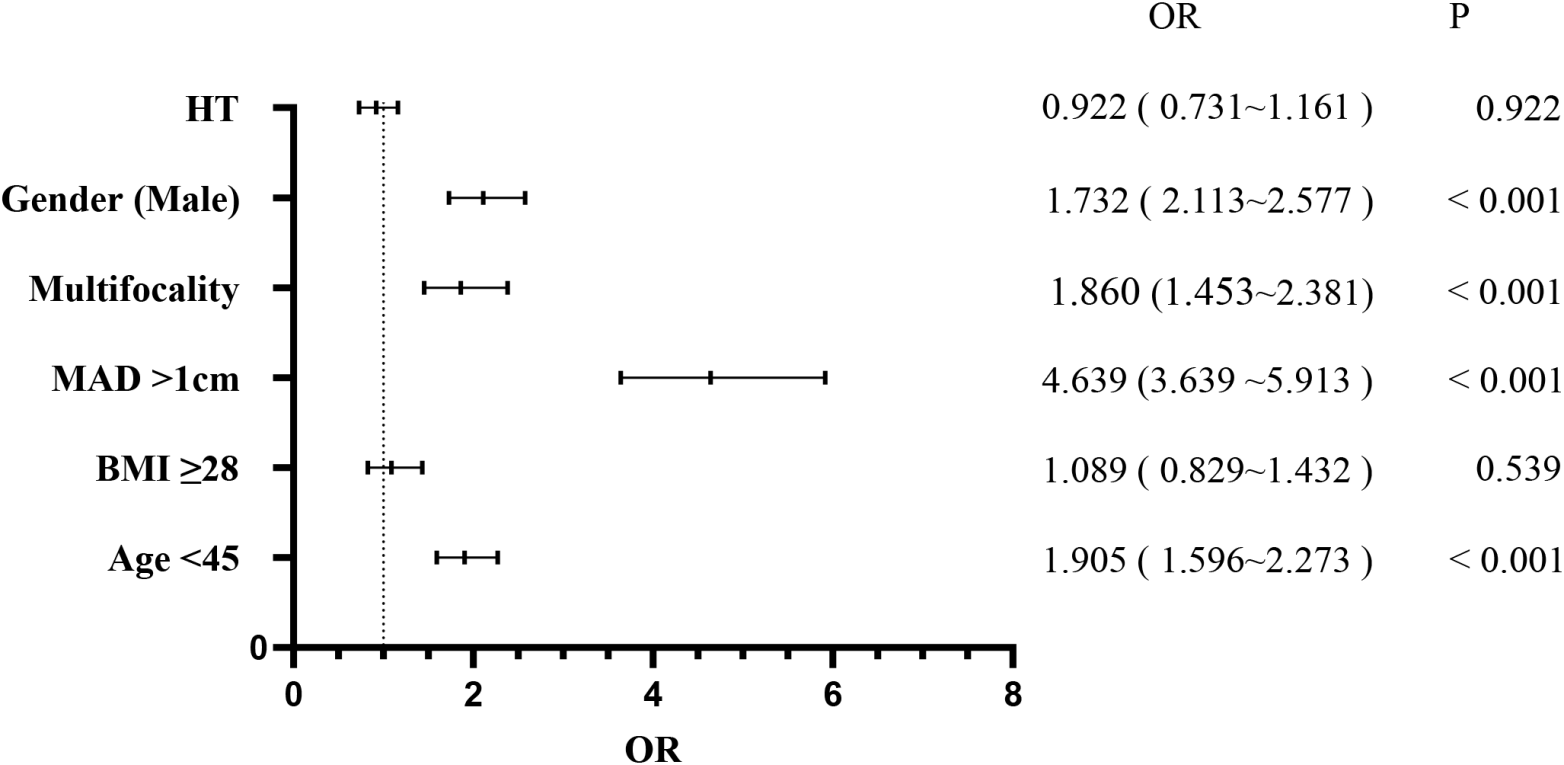
Multivariate logistic regression analysis of central lymph node metastasis.

Patients were stratified into the CLNM+ group (944 patients) and the CLNM- group (1,484 patients). Univariate analysis revealed significant associations between CLNM and gender (P < 0.001), age (P < 0.001), MAD (P < 0.001), BMI (P < 0.001), multifocality (P < 0.001), and HT (P = 0.038) (**Table 1**). Male patients, patients younger than 45 years, patients with MAD > 1 cm, BMI ≥ 28 kg/m², and multifocality were more likely to have CLNM, whereas patients with HT had a lower risk. Multivariable binary logistic regression analysis showed that male gender (OR, 1.732; 95% CI, 2.113–2.577; P < 0.001), age < 45 years (OR, 1.905; 95% CI, 1.596–2.273; P < 0.001), MAD > 1 cm (OR, 4.639; 95% CI, 3.639–5.913; P < 0.001), and multifocality (OR, 1.860; 95% CI, 1.453–2.381; P < 0.001) were independent risk factors for CLNM (**Figure 1**). HT (OR, 0.922; 95% CI, 0.731–1.161; P = 0.922) and BMI (OR, 1.089; 95% CI, 0.829–1.432; P = 0.539) showed weaker associations with CLNM. The Hosmer and Lemeshow Test (P = 0.792) indicated a good fit for the regression model.

**Table 1:** Univariate analysis of the clinical and pathological factors associated with CLNM.

		CLNM		p-value
		+ (n=944)	- (n=1484)	
Gender				
Male	639	338	301	<0.001
Female	1789	606	1183	
Age (years)				
<45	1034	495	539	<0.001
≥45	1394	449	945	
MAD (cm)				
≤1	2039	673	1366	<0.001
>1	389	271	118	
BMI (kg/m^2^)				
≥28	284	136	148	<0.001
<28	2144	808	1336	
Multifocality				
Yes	337	172	165	<0.001
No	2091	772	1319	
HT				
Yes	443	153	290	0.038
No	1985	791	1194	

CLNM, Central lymph node metastasis; MAD, Maximal axial diameter; BMI, Body mass index; HT, Hashimoto’s thyroiditis.

### 3.2 LLNM

**Figure 2:**
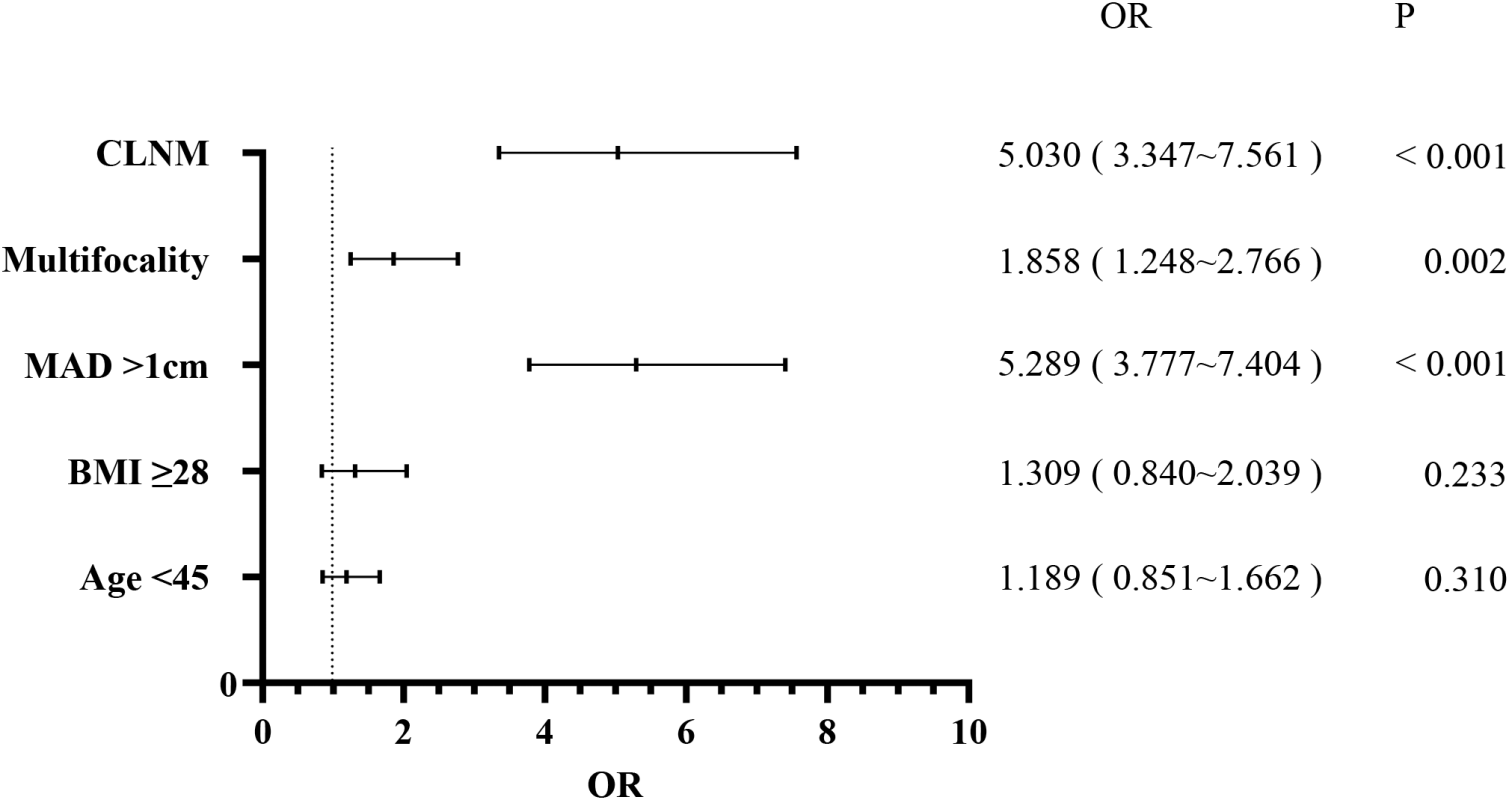
Multivariate logistic regression analysis of lateral lymph node metastasis.

Patients were also categorized into the LLNM+ group (177 patients) and the LLNM- group (2,251 patients). Univariate analysis identified significant correlations between LLNM and age (P = 0.002), MAD (P < 0.001), BMI (P = 0.003), multifocality (P < 0.001), and CLNM (P < 0.001) (**Table 2**). HT (P = 0.794) and gender (P = 0.136) showed no association with LLNM. Patients younger than 45 years, with MAD > 1 cm, BMI ≥ 28 kg/m², multifocality, and CLNM exhibited a higher likelihood of]LLNM. Multivariate binary logistic regression analysis indicated that MAD > 1 cm (OR, 5.289; 95% CI, 3.777–7.404; P < 0.001), multifocality (OR, 1.858; 95% CI, 1.248–2.766; P = 0.002), and CLNM (OR, 5.030; 95% CI, 3.347–7.561; P < 0.001) were independent risk factors for LLNM (**Figure 2**). Age (OR, 1.189; 95% CI, 0.851–1.662; P = 0.310) and BMI (OR, 1.309; 95% CI, 0.840–2.039; P = 0.233) showed weaker associations with LLNM. The Hosmer and Lemeshow Test (P = 0.648) indicated a good fit for the regression model.

**Table 2:** Univariate analysis of the clinical and pathological factors associated with LLNM.

		LLNM		p-value
		+ (n=177)	- (n=2251)	
Gender				
Male	639	55	584	0.136
Female	1789	122	1667	
Age (years)				
<45	1034	95	939	0.002
≥45	1394	82	1312	
MAD (cm)				
≤1	2039	78	1961	<0.001
>1	389	99	290	
BMI (kg/m^2^)				
≥28	284	33	251	0.003
<28	2144	144	2000	
Multifocality				
Yes	337	42	295	<0.001
No	2091	135	1956	
HT				
Yes	443	31	412	0.794
No	1985	146	1839	
CLNM				
+	944	147	797	<0.001
-	1484	33	1451	

CLNM, Central lymph node metastasis; LLNM, Lateral lymph node metastasis; MAD, Maximal axial diameter; BMI, Body mass index; HT, Hashimoto’s thyroiditis.

### 3.3 Tumor location and LNM

Tumor location was categorized as upper, middle, and lower (excluding multi-site distribution tumors and isthmic tumors, with a total of 2,020 patients included). Chi-square tests and multiple comparisons (**Table 3**) indicated that lower pole tumors were more likely to have CLNM compared to upper pole tumors (P = 0.011), while upper pole tumors were more prone to LLNM than lower pole tumors (P = 0.001). Due to multiple comparisons among the three groups, a P-value < 0.0167 was considered statistically significant. In a subset of 149 patients with LLNM (**Table 4**), upper pole tumors showed a higher propensity for skip metastasis (LLNM without CLNM) compared to those located in the middle (P = 0.007) or lower pole (P = 0.016).

**Table 3:** Univariate analysis of the tumor location associated with CLNM and LLNM.

	CLNM		p-value	LLNM		p-value
	+	-		+	-	
Location						
Upper	195	378	0.021	59	514	0.004
Middle	237	431		45	623	
Lower	318	461		44	735	
multiple comparison						
Upper	195	378	0.594	59	514	0.024
Middle	237	431		45	623	
Upper	195	378	0.011	59	514	0.001
Lower	318	461		44	735	
Middle	237	431	0.037	45	623	0.390
Lower	318	461		44	735	

CLNM, Central lymph node metastasis; LLNM, Lateral lymph node metastasis.

**Table 4:** Univariate analysis of the tumor location in relation to PTC with LLNM but no CLNM.

	CLNM		p-value
	+	-	
Location			
Upper	39	20	0.006
Middle	40	5	
Lower	39	6	
multiple comparison			
Upper	39	20	0.007
Middle	40	5	
Upper	39	20	0.016
Lower	39	6	
Middle	40	5	0.748
Lower	39	6	

CLNM, Central lymph node metastasis; LLNM, Lateral lymph node metastasis.

## 4 Discussion

The prognosis for PTC patients is generally favorable, with a low recurrence rate. However, conflicting studies exist regarding the impact of LNM on survival. Some suggest that PTC patients with LNM at initial surgery or experiencing LNM recurrence have a lower survival rate (17–20), while others find no significant effect (21, 22). In most countries, routine lateral lymph node dissection (LLND) occurs only when clear evidence of LNM is present. Prophylactic lymphadenectomy is not recommended for patients without preoperatively detected cervical LNM. At our hospital, CLND is routinely performed for most PTC patients. LLND is only performed when LLNM is confirmed by preoperative or intraoperative pathological examination. Despite controversy over the prognostic significance of lymph node involvement at initial surgery, regional lymph node recurrence remains a concern (23, 24). Considering patients’ quality of life, the presence of LNM plays a crucial role in mitigating subsequent surgical interventions.

Preoperative assessment of LNM in PTC relies on color Doppler ultrasound (US) and neck computerized tomography (CT). However, US sensitivity for detecting LNM is only 60-70% (25, 26), leading to challenges in distinguishing reactive lymphadenopathy from true metastatic disease. Enhanced CT scans can identify calcified metastatic nodes but have limitations in detecting non-calcified nodes (27). Clinicians still struggle to determine the need for LND before PTC surgery.

Our study analyzed risk factors associated with CLNM, LLNM, and skip metastasis. We found that male gender, age <45 years, MAD >1 cm, BMI ≥28 kg/m², and multifocality correlated with CLNM. Interestingly, HT emerged as a protective factor for CLNM. Among these factors, male gender, age <45 years, and MAD >1cm emerged as independent risk factors. While previous studies also linked age <45, male gender, tumor size, and multifocality to CLNM (28–30). Some studies indicated HT as a risk factor for CLNM (28), but others suggested potential protective mechanisms of HT (31). The specific impact of HT remains unclear. Our study showed a reduction in the risk of CLNM occurrence in HT patients. Close monitoring for CLNM is recommended in patients with these independent risk factors, considering prophylactic CLND.

The variables associated with the incidence of LLNM included age < 45 years, MAD > 1 cm, BMI ≥ 28 kg/m², multifocality, and CLNM. Meanwhile, MAD > 1 cm, multifocality, and CLNM established as independent risk factors for LLNM. This study also revealed that tumors situated in the upper pole exhibited a higher propensity for developing LLNM and skip metastasis, while those located in the lower pole were more likely to develop CLNM. A meta-analysis suggested that CLNM, tumor size, multifocality, male gender, and tumor location were independent risk factors for LLNM (32), which aligns with the results of this study. Zhao et al. (33) also identified a tumor diameter > 1 cm and CLNM as independent risk factors for LLNM, and found that upper pole tumors had a higher propensity for skip metastasis, consistent with our findings. Upper pole tumors are more likely to demonstrate skip metastasis, potentially due to preferential dissemination of the tumor via the superior thyroid artery to the lateral lymph nodes rather than through the lymphatic vessels (34).We recommend that PTC patients with preoperative examinations indicating tumor size > 1 cm, multiple lesions, or evident CLNM found intraoperatively, should be carefully evaluated for the potential presence of LLNM. These individuals may undergo lateral lymph node (LLN) biopsy during surgery, and if deemed necessary, LLND may be performed. For tumors located at the upper pole, even without apparent CLNM, monitoring of LLN should still be conducted.

Furthermore, BMI was a key variable of interest in this investigation. Given the observed higher incidence of LNM among obese patients in clinical practice, we conducted a comparison between obese patients (BMI ≥ 28 kg/m²) and non-obese patients (BMI < 28 kg/m²). Univariate analysis revealed that obese patients exhibited a greater likelihood of CLNM and LLNM compared to non-obese patients. However, multivariate analysis indicated that BMI did not independently contribute to the risk of LNM. The relationship between obesity and LNM remains ambiguous, with limited relevant studies available (35). Inclusion of BMI in the analysis provided clinicians valuable insights.

Although numerous studies have investigated the risk factors for CLNM or LLNM, the conclusion is still controversial. Our study conducted a comprehensive statistical comparison of the risk factors associated with CLNM, LLNM, and skip metastasis, offering a more thorough analysis. LNM significantly contributes to postoperative recurrence and poor prognosis in patients with PTC, potentially necessitating secondary surgery and impacting patients’ psychological well-being and quality of life. However, performing LND on all patients may increase the incidence of unnecessary surgical complications such as recurrent laryngeal nerve injury, hypoparathyroidism, and chylothorax. Consequently, preoperatively evaluating LNM and determining whether to perform LND has remained a challenging issue for surgeons. Some studies have indicated that prophylactic CLND can reduce local recurrence rates in high-risk patients (36, 37), while therapeutic LLND should be considered for those with suspected or confirmed LLNM preoperatively (14). US and CT imaging have inherent limitations in distinguishing between different types of lymph nodes, and biopsy is not routinely performed as an examination method. When combined with an analysis of LNM risk factors, these imaging methods can enhance effectiveness and accuracy in evaluating lymph nodes, reducing the likelihood of misdiagnosis, ultimately diminishing the risk of recurrence, and enhancing patient quality of life.

Our study has certain limitations. Firstly, it is a retrospective study, and despite the large sample size, the results may be subject to bias. Secondly, there is a possibility of occult cancer or LNM that cannot be diagnosed through pathology. Thirdly, due to the limited number of skip metastasis cases, we did not conduct a multivariate analysis in this subgroup. Therefore, we hope that future research will involve a multi-center prospective study to more accurately analyze the risk factors for PTC patients.

## 5 Conclusion

Despite the overall postoperative recurrence rate in PTC patients is low, identifying risk factors such as male gender, age < 45 years, MAD > 1 cm, multifocality, and CLNM can help predict LNM. In specific cases, selective lymphadenectomy in the central or lateral neck area may be warranted.

## Data Availability

The original contributions presented in the study are included in the article/**Supplementary Material**. Further inquiries can be directed to the corresponding author.
